# CD4^+^ T cell activation and associated susceptibility to HIV‐1 infection in vitro increased following acute resistance exercise in human subjects

**DOI:** 10.14814/phy2.14234

**Published:** 2019-09-25

**Authors:** Alexander K. Holbrook, Hunter D. Peterson, Samantha A. Bianchi, Brad W. Macdonald, Eric C. Bredahl, Michael Belshan, Jacob A. Siedlik

**Affiliations:** ^1^ Department of Medical Microbiology and Immunology Creighton University Omaha Nebraska; ^2^ Department of Exercise Science and Pre‐Health Professions Creighton University Omaha Nebraska

**Keywords:** Physical activity, T cell activation, viral infection

## Abstract

Early studies in exercise immunology suggested acute bouts of exercise had an immunosuppressive effect in human subjects. However, recent data, show acute bouts of combined aerobic and resistance training increase both lymphocyte activation and proliferation. We quantified resistance exercise‐induced changes in the activation state of CD4^+^ T lymphocytes via surface protein expression and using a medically relevant model of infection (HIV‐1). Using a randomized cross‐over design, 10 untrained subjects completed a control and exercise session. The control session consisted of 30‐min seated rest while the exercise session entailed 3 sets × 10 repetitions of back squat, leg press, and leg extensions at 70% 1‐RM with 2‐min rest between each set. Venous blood samples were obtained pre/post each session. CD4^+^ T lymphocytes were isolated from whole blood by negative selection. Expression of activation markers (CD69 & CD25) in both nonstimulated and stimulated (costimulation through CD3^+^CD28) cells were assessed by flow cytometry. Resistance exercised‐induced effects on intracellular activation was further evaluated via in vitro infection with HIV‐1. Nonstimulated CD4^+^ T lymphocytes obtained postexercise exhibited elevated CD25 expression following 24 h in culture. Enhanced HIV‐1 replication was observed in cells obtained postexercise. Our results demonstrate that an acute bout of resistance exercise increases the activation state of CD4^+^ T lymphocytes and results in a greater susceptibility to HIV‐1 infection in vitro. These findings offer further evidence that exercise induces activation of T lymphocytes and provides a foundation for the use of medically relevant pathogens as indirect measures of intracellular activation.

## Introduction

The human immune system is divided into two categories, innate and adaptive, that can elicit both broad or highly targeted responses. CD4^+^ T cells are essential mediators of both the innate and adaptive immune responses. They play an integral role in the overall immunocompetence of an individual through the release of cytokines and chemokines, the recruitment of immune cells to sites of infection and inflammation, the activation of macrophages, and the activation of B cells to produce antigen‐specific antibodies. In the adaptive arm, the activation of CD4^+^ T cells following exposure to cognate antigen (Medzhitov 2007) is associated with changes in expression of specific proteins, including increased expression of CD69 and CD25, which are used as early (Testi et al. 1989) and intermediate (Malek 2008) markers of cellular activation, respectively.

It has been demonstrated that both aerobic and resistance exercise have the potential to alter the immune state of an individual. Evidence from a 2016 meta‐analytic review suggests the proliferative response of mixed lymphocyte populations (i.e., peripheral blood mononucleocytes (PBMCs)) to in vitro mitogenic stimuli is suppressed following an acute bout of exercise (Siedlik et al. 2016). Notably, most of the research investigating the effect of exercise on immunity have focused on PBMC proliferation in response to an acute bout of aerobic exercise (Walsh et al. 2011; 2011). In comparison, limited research has been conducted examining the relationship between acute bouts of resistance training and immunity often with conflicting results (Dohi et al. 2001; Koch et al. 2001; Potteiger et al. 2001; Chan et al. 2003). Nieman et al. (Nieman et al. 1995) observed no significant differences in concanavalin A (ConA) stimulated lymphocyte proliferation in trained men following repeated sets of 10 repetition back squats at 65% of 1 repetition maximum (1RM). Potteiger et al. (2001), however, observed reduced phytohemagglutinin (PHA) stimulated lymphocyte proliferation in untrained females following an acute bout of lower limb resistance training, but no change in proliferation for trained female participants. As seen above, the training status of the participants, as well as the mitogens used for stimulation, may affect in vitro proliferative responses. Moreover, the question of how different immune subsets respond to acute resistance exercise remains unanswered, preventing a full understanding of the clinical consequence of exercise prescription.

Cellular activation in response to an antigen leads to clonal expansion of antigen‐specific T cells to facilitate neutralization of an invading pathogen (Mueller et al. 1989). In laboratory and clinical settings, clonal expansion in response to either mitogenic stimulation or co‐stimulation through CD28 is commonly utilized as a measure of lymphocyte functional ability (Siedlik et al. 2016; 2017). Indeed, studies investigating the effects of exercise on the immune system have commonly used proliferation assays to quantify changes in lymphocyte function following an acute bout of aerobic exercise. As addressed briefly above, however, proliferative assays can produce ambiguous results due to methodological variability (Siedlik et al. 2016) and a failure to quantify specific elements of the activation process. Measurement of specific immune cell functions potentially can improve the understanding of the impact of excise on immunity.

Exercise‐induced alterations in immune function have the potential to affect the interaction of pathogens with the immune system. A change in cellular activation state and/or subsequent alteration in proliferation of immune cell subsets could alter the disease state in an individual. For example, changes in CD4^+^ T lymphocyte activation could enhance an immune response and provide greater protection against an invading pathogen. Conversely, heightened cell activation may create favorable conditions for lymphotropic pathogens, such as human immunodeficiency virus (HIV). Type 1 HIV (HIV‐1), the etiologic agent of acquired immune deficiency syndrome (AIDS), is a member of the retrovirus family that preferentially infects activated CD4^+^ T lymphocytes. More than 36 million persons are infected with HIV‐1 worldwide (UNAIDS). HIV‐1 replication correlates strongly with the activation state of T cells due to the metabolic requirements of reverse transcription and integration into the host genome (Gowda et al. 1989; Stevenson et al. 1990). Hence, any transient alteration in the metabolic state of a CD4^+^ T cell will change its susceptibility to HIV‐1. Here, we propose to use HIV‐1 infection as a biological model to independently asses CD4^+^ T cell activation state. Notably, quiescent CD4^+^ T cells, which are metabolically and transcriptionally silent (Yusuf and Fruman 2003; Tzachanis et al. 2004), were originally thought to be refractory to HIV infection; however, evidence suggests that partially activated cells can support infection, especially with subsequent stimulation (Stevenson et al. 1990; Zack et al. 1990, 1992; Korin and Zack, 1998; Unutmaz et al. 1999; Manganaro et al. 2018).

Previous work from our laboratory demonstrated an increase in CD25 expression and enhanced proliferative responses in CD4^+^ T cells following combined aerobic and resistance training exercise (i.e., circuit training) (Siedlik et al. 2017). In this study, we focused our efforts to investigate whether resistance training alone altered CD4^+^ T cell activation state and response to co‐stimulation with CD28. In parallel we tested susceptibility to HIV‐1 infection to independently validate any changes in activation state resulting from exercise. To do this, we compared CD4^+^ T cells isolated from individuals prior to and after acute resistance training. Notably, the data were cross‐compared to a nonexercise session as an additional control. Changes in early markers of activation, specifically expression of CD25 and CD69, were analyzed from two distinct perspectives. First, was there an exercise‐induced effect on CD4^+^ T cell activation absent stimuli in culture (Arm 1), and second, was there an exercise‐induced effect on the ability of CD4^+^ T cells to respond to stimuli in culture (Arm 2). Together, this study represents a first attempt to quantify exercise‐induced changes in CD4^+^ T cell function using a medically relevant viral model.

## Methods

### Participants

Ten healthy, untrained, college‐aged individuals (Mean ± SD, *n* = 10 [5 male & 5 female], age = 21 ± 2 year; weight = 71.5 ± 10.1 kg; height = 171.8 ± 7.1 cm) volunteered to participate. Untrained status was defined as no more than 1 h of aerobic and/or resistance training per week. All participants provided written informed consent and completed a Health & Exercise Status Questionnaire prior to participation. At the time of recruitment, subjects were instructed to maintain their normal dietary patterns prior to participating in either session, but to refrain from exercise for 24 h prior to data collection. Participants reported that they had neither recently taken nor were currently using non‐steroidal anti‐inflammatory drugs (NSAID), aspirin, or other anti‐thrombotic over‐the‐counter or prescription medications. All participants reported being negative for HIV infection. Participants reported no cold or flu symptoms in the 2 weeks prior to data collection. The study conformed to the standards set by the Declaration of Helsinki and the procedures followed were in accordance with the protocol approved by the Creighton University Institutional Review Board (959210‐1).

### 3‐repetition maximum assessments

Each participant completed an initial strength assessment that included 3‐repetition maximum [RM] barbell high bar parallel squat, 3‐RM leg press, and 3‐RM leg extension. The 3‐RM back squat was tested on their first visit and the leg press and leg extension were assessed at a visit at least 2 days after squat testing. All participants completed a 5 min self‐paced warm‐up on a cycle ergometer prior to starting any warm‐up sets. The 3‐RM testing followed the protocol recommended by the National Strength and Conditioning Association (Haff and Triplett 2016). For all exercises, the first warm‐up set required 5–10 repetitions. The second warm‐up set and beyond required 2–5 repetitions until a 3‐RM was attempted. Participants were allowed multiple attempts at the 3‐RM to attain the highest load possible. The 3RM was recorded and used for estimation of 1RM values using the following equation: (3RM/ 0.9 = 1RM). All experiment visits took place at least 1 week after completion of repetition maximum testing.

### Testing protocol

Following the fitness assessments, each participant randomly completed a control and exercise session, which occurred between 0700 and 0745 h. Both visits occurred within a 7‐day time frame. The exercise session included a 5‐min self‐paced warm‐up on a cycle ergometer followed by 3 sets of 10 repetitions of back squats at 70% of estimated 1‐RM, 3 sets of 10 repetitions of leg press at 70% 1‐RM, and 3 sets of 10 repetitions of leg extension at 70% 1‐RM with 2‐min rest between sets. The control session involved the subjects sitting quietly in a room for 30 min. Subjects were not allowed to read or use electronic devices during this time and were monitored at random intervals to ensure they remained alert.

### Physiological monitoring

Participants were fitted with a Zephyr BioHarness 3 (Zephyr Technology, Annapolis, MD) to measure heart rate (HR). Continuous HR measures were recorded at 1 sec intervals during the training session and downloaded using the Zephyr BioHarness Log Downloader (version 1.0.29.0). HR_max_ was estimated using the methods of Tanaka et al. (2001).

### Blood collections

Blood samples were collected in sodium heparin vacutainers prior to (Pre) and immediately following (Post) each testing session using standard antecubital venipuncture technique.

### Antibodies and reagents

Antibodies used for flow cytometry were purchased from BioLegend (San Diego, CA) and include: anti‐CD4‐Alexa Fluor 700 (RPA‐T4), CD69‐APC/Cy7 (FN50), and CD25‐PE (M‐A251).

### Cell purification and culture

Blood samples (80 mL) were obtained at each time point for analyses of surface marker expression and viral replication. CD3^+^CD4^+^ T cell isolation from peripheral blood was conducted through negative selection using a Human CD4^+^ T cell enrichment kit as directed by the manufacturer (Stemcell Technologies, Vancouver, BC, Canada). Purity was assessed following cell isolation by staining with anti‐CD4‐Alexa Fluor 700 (RPA‐T4) and all samples were >97% CD4^+^ by flow cytometry (Kohlmeier et al. 2006; Newton and Benedict, 2014; Siedlik et al. 2017). After purification, the cells were resuspended in warm Immunocult‐XF T cell expansion medium (Stemcell Technologies, Vancouver, BC, Canada).

### Surface marker expression – cell stimulation and culture

**Figure 1:**
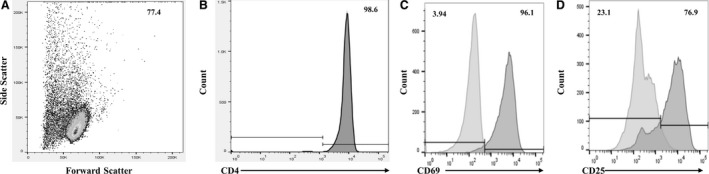
Representative gating procedures for analyzed samples. (A) Illustrates usage and placement of the live cell gate in the forward scatter versus side scatter plot. Sample populations were all >97% CD3^+^CD4^+^ following isolation. All flow data were gated as in (A) before further analysis. An unstained sample (not shown) was used as a guide for placement of the CD4^+^ gate in the fully stained sample (B). Expression of surface markers of activation in non‐stimulated and stimulated cell populations were quantified using median fluorescent intensity (MFI) of (C) CD69 and (D) CD25. Overlays of the non‐stimulated (light gray) and stimulated samples (dark gray) were used to correct for the effect of costimulation through CD28 prior to analyzing exercise‐induced alterations.

Cells were co‐stimulated through CD3^+^CD28 using plate‐bound antibodies or no simulation as previously described (Chirathaworn et al. 2002; Kohlmeier et al. 2006; Siedlik et al. 2017). Each antibody was titrated to the lowest concentration that gave maximum T cell activation: anti‐CD3 (OKT3) used at 1 *μ*g/mL (BioLegend, San Diego, CA) and anti‐CD28 (CD28.2) at 2 *μ*g/mL (BioLegend, San Diego, CA). Antibodies were diluted in sterile Dulbecco’s Phosphate Buffered Saline (dPBS) (Life Technologies, Grand Island, NY) added to 96‐well plates and incubated overnight at 4°C. Unbound antibodies were removed by washing 3x with dPBS prior to cell plating. CD4^+^ T cells were plated at 1.5 × 10^6^ cells/mL in 200 *μ*L of Immunocult‐XF T‐cell media (Stemcell Technologies, Vancouver, BC, Canada) directly after isolation. Cells were cultured at 37°C in a humidified incubator with 5% CO_2_. Cells were analyzed by flow cytometry using anti‐CD4‐Alexa Fluor 700, anti‐CD69‐APC/Cy7, and anti‐CD25‐PE antibodies immediately after CD4^+^ T cell isolation (0 h), 24 h, and 72 h in culture using a ZE5 Cell Analyzer (Propel Labs, Fort Collins, CO). Data analysis was performed with FlowJo software v10 (TreeStar, Ashland, OR). Compensation was performed using single antibody positive and negative controls (OneComp eBeads Compensation Beads, ThermoFisher Scientific, Waltham, MA) in each assay. Gates were set based on fluorescence minus one controls. A representative gating strategy is shown in Figure 1.

### HIV‐1 viral replication assays ‐ cell stimulation and culture

**Figure 2:**
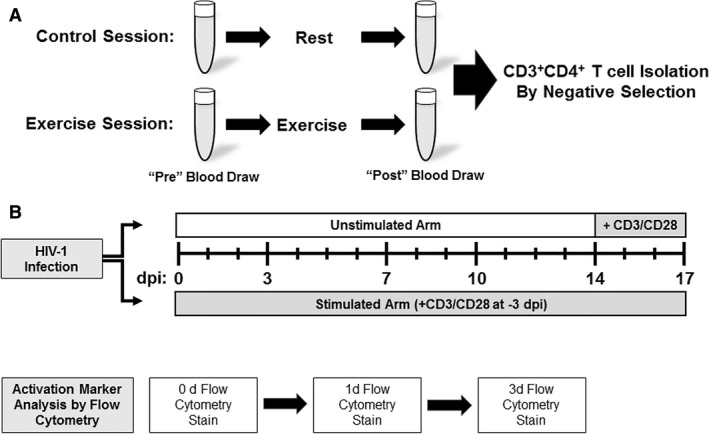
Overview of experiment design. Each subject participated in both a control and exercise session with order randomized. Blood was collected pre and post each visit (A) and CD3^+^CD4^+^ T cells isolated via negative selection. (B) Both unstimulated and CD3/CD28 stimulated cells were cultured for 17 days. Stimulated CD4^+^ T cell HIV‐1 infections occurred after 3 days of CD3/CD28 stimulation. Supernatant samples were collected at 0, 3, 7, 10, 14, and 17 days post infection (dpi). Surface protein expression was quantified via flow cytometry at 0, 1, and 3 days.

HIV‐1 NLX virus stocks were produced through the transfection of 293T cells with 5 *μ*g of pNLX molecular clone and quantified by p24 antigen ELISA as previously described (Siedlik et al. 2016). Both unstimulated and CD3/CD28 stimulated cells were infected for each condition. Unstimulated CD4^+^ T cells were infected and cultured shortly after purification. 1 × 10^6^ cells were seeded in three wells as per condition in a 24‐well plate and incubated with HIV‐1 at a multiplicity of infection (MOI) of 0.1 for 4 h at 37°C with 5% CO_2_. Cells were then pelleted, washed with dPBS, and resuspended in 550 *μ*L fresh culture media (RPMI‐1640 Medium [GE Healthcare, Piscataway, NJ] + 10% Fetal bovine serum [Corning, Corning, New York] + 2% Penicillin/Streptomycin [Corning, Corning, New York] + 20 mmol/L L‐glutamine [Corning, Corning, New York] + 50 units/mL recombinant Human IL‐2 [R&D Systems, Minneapolis, MN]) per well, in a new 24‐well plate. Cells were stimulated using plate‐bound antibodies as outlined above, but in 24‐well plates at 2 × 10^6^ cells/mL in 500 *μ*L of Immunocult‐XF T‐cell media (Stemcell Technologies, Vancouver, BC, Canada) culture. Stimulated CD4^+^ T cell HIV‐1 infections occurred after 3 days of CD3/CD28 stimulation. Both unstimulated and CD3/CD28 stimulated cells were cultured for 17 days. Supernatant samples were collected at 0, 3, 7, 10, 14, and 17 days post infection (dpi), clarified by centrifugation, and stored at −20°C. For the reactivation studies, unstimulated cells were activated at 14 dpi with human CD3/CD28/CD2 T cell activator beads for 3 days (Stemcell Technologies, Vancouver, BC, Canada) and additional RT sample collected at 17 dpi. An overview of the cell culture experiments is shown in Figure 2.

### Reverse transcriptase assay

Virus replication/production was quantified by a reverse transcriptase (RT) assay as described previously (DeBoer et al. 2018). Triplicate 10 μL of supernatant were assayed per timepoint. Fresh culture media was used as a negative control, and an NLX virus standard as a positive control in each reaction plate. An RT assay mix of H_2_0, 50 mmol/L Tris (pH 7.9), 75 mmol/L KCL, 2 nmol/L DTT, 0.1875 mmol/L ATP, 5 mmol/L MgCl_2_, RT Primer (25 mg/L), 0.05% NP‐40, 2 *μ*mol/L dTTP, and 2 *µ*Ci [^32^P]‐*α*‐TTP was prepared and vortexed thoroughly (Siedlik et al. 2016). 30 *μ*L of RT assay mix was added to each well and the plate was incubated at 37°C for 3 h. RT mix was added to Whatman paper in individual spots and allowed to dry. The paper was washed three times with 2× saline‐sodium citrate (SSC) for 5 min while rocking, washed once with 95% Ethanol, and allowed to dry. The dried paper was then exposed to a phosphor screening plate. After overnight exposure, the phosphor plates are analyzed on GE Amersham Molecular Dynamics Typhoon 9410 Molecular Imager v5.0 (GE Healthcare, Piscataway, NJ). The image was quantified using Molecular Dynamics ImageQuant v5.2 software (GE Healthcare, Piscataway, NJ), setting the background to a negative control sample.

### Statistical analysis

Power calculations were based on previously reported data that investigated exercise‐induced changes in T cell activation and proliferation (Siedlik et al. 2017). The power analysis indicated 10 subjects would exceed 80% power for detecting an effect size of *d_z_* = 0.99 [a large effect as outlined by Cohen (1992)] for relevant differences at an alpha < 0.05. For this investigation, quantification of the change in values from pre‐to‐post was of more interest than the absolute values of the measurements themselves; therefore, fold change scores were calculated using log2 transformations: log2(Post/Pre). Normality of data was verified using the D’Agostino‐Pearson test. Data were analyzed using paired samples *t*‐tests in R version 3.3.1 (Team 2014). Given the exploratory nature of this project, Bayesian paired samples *t*‐tests were also performed using the BEST package in R (Kruschke 2013). Reported parameter estimates from Bayesian models include the posterior mean difference, 95% highest density intervals (HDI), probability the true mean difference is greater than 0, and, when relevant, threshold estimates to determine the probability the true difference of the means is greater than a 10% increase.

## Results

### Resistance training session induced substantial changes in heart rate

The exercise session elicited a substantial sympathetic stimulus as demonstrated by the HR data. The mean predicted HR_max_ for all subjects was 193 ± 2 bpm. Average HR during the exercise trial was 137 ± 14 bpm compared with 82 ± 10 bpm in the control subjects (*P* < 0.001). The average peak HR during the trials was 174 ± 14 bpm and 109 ± 11 bpm for exercise and control, respectively (*P* < 0.001). During the exercise session, subjects spent approximately half of the time (13.4 ± 7.3 min) working at over 70% of their predicted HR_max_, whereas all subjects spent the entirety of the control session under the 60% threshold of predicted HR_max_. Summary of statistical results and comparative analyses are presented in Table 1.

**Table 1:** Summary output for all statistical analyses performed

	Null hypothesis significance test						Bayesian analyses			
	Time	Mean_diff_	*t*	df	*P*	95% CI	Post. Mean_diff_	SD_diff_	95% HDI	*P* (Mean_diff_ > 0)
No‐Stim										
CD25	0 h	−0.2	−0.73	9	0.49	−0.8, 0.41	−0.2	0.89	−0.84, 0.47	0.25
	24 h	0.27	2.4	9	0.04	0.02, 0.52^*^	0.28	0.37	0.01, 0.53	0.98
	72 h	0.04	0.12	9	0.91	−0.7, 0.78	0.06	1.1	−0.74, 0.83	0.57
CD69	0 h	−0.29	−0.74	9	0.48	−1.7, 0.6	−0.23	1.3	1.2, 0.71	0.29
	24 h	0.02	0.09	9	0.93	−0.37, 0.41	0.02	0.58	−0.41, 0.43	0.55
	72 h	0.1	0.36	9	0.73	−0.53, 0.74	0.11	0.93	−0.56, 0.77	0.65
CD4	0 h	−0.04	−1.41	9	0.19	−0.1, 0.02	−0.04	0.1	−0.11, 0.03	0.13
	24 h	0.02	0.32	9	0.76	−0.11, 0.15	0.02	0.19	−0.12, 0.16	0.62
	72 h	0.05	1.09	9	0.3	−0.05, 0.14	0.05	0.15	−0.06, 0.15	0.82
Stim										
CD25	24 h	−0.58	−0.75	9	0.47	−2.35, 1.18	−0.51	2.5	−2.4, 1.3	0.27
	72 h	0.95	2.08	9	0.07	−0.08, 1.98	0.96	1.5	−0.16, 2.1	0.96
CD69	24 h	−0.36	−0.8	9	0.45	−1.37, 0.65	−0.04	0.76	−0.94, 0.57	0.43
	72 h	0.22	0.76	9	0.47	−0.43, 0.86	0.23	0.96	−0.48, 0.9	0.76
CD4	24 h	−0.06	−0.12	9	0.91	−1.24, 1.12	−0.29	1.3	−1.2, 0.75	0.26
	72 h	0.15	0.2	9	0.84	−1.49, 1.78	0.12	2.4	−1.6, 2	0.56
HIV‐1										
Viral replication	0 day	0.35	1.34	9	0.21	−0.25, 0.97	0.21	0.6	−0.23, 0.78	0.87
	3 day	0.28	2.68	9	0.03	0.04, 0.51^*^	0.27	0.35	0.02, 0.53	0.98
	7 day	0.3	0.79	9	0.44	−0.56, 1.15	0.31	1.3	−0.65, 1.2	0.76
Heart rate										
Average		56	13.14	9	<0.001	46, 65^*^	56	14	46, 66	>0.999
Peak		66	12.42	9	<0.001	54, 78^*^	65	17	52, 77	>0.999

Mean differences were calculated as Experiment – Control.

### Exercise induced changes in expression of CD25 but not CD69

**Figure 3:**
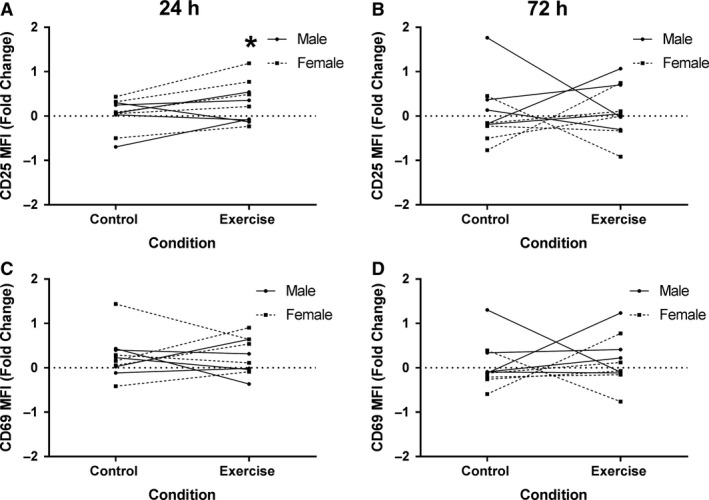
Expression of surface markers of activation increased on non‐stimulated CD4^+^ T cell populations. Human CD4^+^ T cells were isolated and cultured in non‐stimulated wells for 1 and 3 days then stained for CD25 and CD69 and analyzed by flow cytometry. The median fluorescence intensity (MFI) was determined for expression of CD25 at (A) 1 day (Control: 0.03 ± 0.36, Exercise: 0.3 ± 0.46) and at (B) 3 days (Control: 0.07 ± 0.7, Exercise: 0.11 ± 0.59). CD69 expression at (C) 1 days (Control: 0.25 ± 0.49, Exercise: 0.27 ± 0.54) and at (D) 3 days (Control: 0.06 ± 0.52, Exercise: 0.16 ± 0.55). Data are presented as fold change from baseline and visualized as spaghetti plots with each line representing the change between paired samples; *n* = 10. * Indicates a statistically significant difference (*P* < 0.05) from the control session.

Surface expression of CD4, CD25, and CD69 was quantified via median fluorescent intensity [MFI] immediately after CD4^+^ T cell isolation (0 h) and after 24 and 72 h in culture. Notably, CD4 expression was not significantly different in cells isolated pre or post the control and exercise session (data not shown). When examining exercise‐induced changes in nonstimulated CD4^+^ T cells (Arm 1), there was no significant difference in CD25 expression on cells isolated before either the control or exercise session (Pre). There was, however, a significant increase in CD25 expression on cells isolated postexercise relative to the control session after 24 h in culture (*P* = 0.04, Fig. 3a). The estimated mean difference using the Bayesian model is 0.28, equivalent to a fold change increase > 21% in the cells collected pre‐to‐post the exercise session compared to the control. Moreover, 95% HDI does not include zero, and the probability the true value is greater than zero is 98% (Table 1). A threshold estimation indicates there is an 86.5% probability that the true difference in means is greater than 10%. The analysis implies that exercise‐induced changes in CD25 expression, in addition to being statistically significant, are likely physiologically relevant. However, after 72 h in culture the effect dissipated (*P* = 0.91, Fig. 3b) indicating that the increased expression of CD25 was transient. No significant differences in the expression of CD69 were observed between the exercise and control sessions at any time points (Fig. 3C–D). Summary data for the intensity of surface protein expression on nonstimulated cells (Arm 1) are shown in Table 2.

**Table 2:** Summary data for surface protein expression on nonstimulated CD4^+^ T lymphocytes

		0 h	24 h	72 h
Control				
CD4	Pre	9578.9 ± 544.7	8966.1 ± 1216.5	9151.3 ± 1184.3
	Post	9868.3 ± 541.7	8975.9 ± 1022.2	9454.5 ± 1270.9
CD69	Pre	203.3 ± 143.2	171.4 ± 84.7	150.8 ± 40.1
	Post	207.0 ± 100.2	198.4 ± 71.4	159.2 ± 52.6
CD25	Pre	342.8 ± 156.9	291.8 ± 102.1	195.3 ± 55.8
	Post	374.3 ± 150.8	291.3 ± 71.6	212.5 ± 83.7
Exercise				
CD4	Pre	9456.9 ± 757.3	9196.3 ± 2251.4	8998.7 ± 1159.1
	Post	9476.2 ± 737.0	9269.2 ± 1850.5	9653.7 ± 1691.0
CD69	Pre	169.7 ± 66.1	172.1 ± 99.2	160.4 ± 56.5
	Post	180.5 ± 99.5	194.2 ± 69.7	173.3 ± 52.6
CD25	Pre	340.9 ± 117.0	325.4 ± 233.3	230.2 ± 90.6
	Post	332.7 ± 123.1	370.1 ± 206.1	231.2 ± 41.0

Values represent median fluorescent intensity (MFI) assessed via flow cytometry. Data are presented as Mean ± SD.

**Figure 4:**
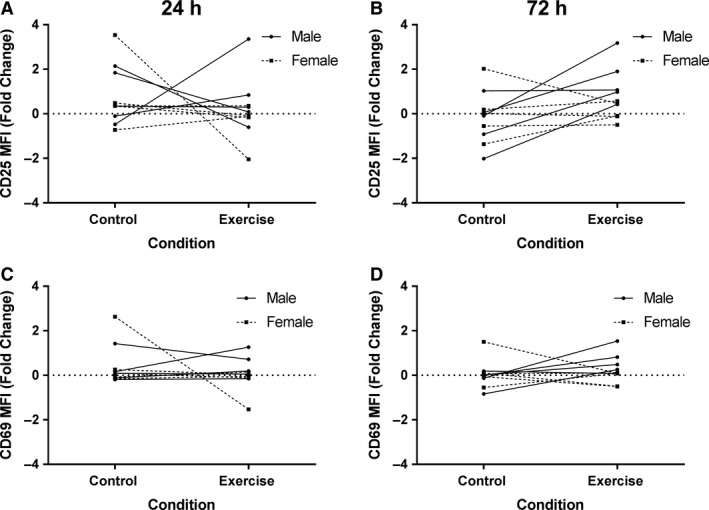
Expression of surface markers of activation in stimulated CD4^+^ T cell populations. Human CD4^+^ T cells were stimulated for 1 and 3 days with anti‐CD3 and anti‐CD28 then stained for CD25 and CD69 and analyzed by flow cytometry. The median fluorescence intensity (MFI) was determined for expression of CD25 at (A) 1 days (Control: 0.77 ± 1.3, Exercise: 0.19 ± 1.3) and at (B) 3 days (Control: −0.16 ± 1.2, Exercise: 0.79 ± 1.1). CD69 expression at (C) 1 days (Control: 0.41 ± 0.91, Exercise: 0.06 ± 0.71) and at (D) 3 days (Control: 0.03 ± 0.61, Exercise: 0.25 ± 0.6). Data are presented as fold change from baseline and visualized as spaghetti plots with each line representing the change between paired samples; *n* = 10.

In Arm 2, we investigated the CD4^+^ T cell response to co‐stimulation through CD28 (Fig. 4A–B). The results showed increased expression of CD25, similar to Arm 1, but no statistically significant (*P* < 0.05) differences were observed. Interestingly, the average fold change in CD25 expression after 72 h in culture was elevated in the exercise session compared to the resting control session, but not to a statistically significant level (*P* = 0.07, Fig. 4B). Follow‐up Bayesian analyses to increase the predictive precision estimated the mean difference for CD25 expression at 72 h as 0.96 (equivalent to an increase > 93% in the exercise session relative to the control). The 95% HDI of the difference of means includes zero, but has 96% of the credible values greater than zero. Thus, there is a strong probability that the estimated parameter is greater than zero (i.e., indicative of an exercise‐induced increase in expression). The probability that the true mean difference would be greater than a 10% increase in CD25 expression was calculated at 93.3%. This suggests there is a 93.3% chance that exercise alters CD4^+^ T cell response to stimuli. Similar to the nonstimulated Arm, there were no significant differences in CD69 expression observed at either 24 h or 72 h (Fig. 4C–D). Data for the intensity of surface protein expression on stimulated cells is summarized in Table 3. Summary data for the percent of activated cells in both stimulated and nonstimulated cultures are shown in Table 4.

**Table 3:** Summary data for surface protein expression on CD4^+^ T cells costimulated through CD28

		24 h	72 h
Control			
CD4	Pre	6092.3 ± 2049.0	15675.9 ± 3537.3
	Post	6302.2 ± 2090.4	17547.6 ± 3360.9
CD69	Pre	4072.4 ± 2930.5	1031.2 ± 269.9
	Post	4436.9 ± 2305.4	1084.5 ± 339.1
CD25	Pre	2850.3 ± 2356.4	5496.5 ± 4290.7
	Post	3364.4 ± 1889.7	4718.2 ± 3469.1
Exercise			
CD4	Pre	6417.7 ± 2726.9	16004.6 ± 5179.2
	Post	6320.8 ± 2505.9	16762.9 ± 5133.9
CD69	Pre	4422.8 ± 2475.5	1748.0 ± 1746.5
	Post	4020.4 ± 2026.4	1721.3 ± 1270.1
CD25	Pre	3539.9 ± 2154.8	5391.2 ± 4791.3
	Post	3819.4 ± 2739.6	7203.3 ± 5967.8

Values represent median fluorescent intensity (MFI) assessed via flow cytometry. Data are presented as Mean ± SD.

**Table 4:** Summary data for percent of CD4^+^ T lymphocytes expressing both CD25 and CD69

		0 h	24 h	72 h
No‐stimulation				
Control	Pre	0.4 ± 0.2	0.6 ± 0.5	0.7 ± 0.3
	Post	0.7 ± 0.7	0.6 ± 0.4	0.7 ± 0.3
Exercise	Pre	0.4 ± 0.2	0.5 ± 0.2	0.8 ± 0.5
	Post	0.6 ± 0.5	0.4 ± 0.2	0.7 ± 0.4
Stimulation through CD3^+^CD28				
Control	Pre		57.9 ± 19.4	69.4 ± 18.3
	Post		64.0 ± 12.7	72.7 ± 14.5
Exercise	Pre		60.5 ± 19.9	68.8 ± 20.0
	Post		61.3 ± 17.9	73.6 ± 15.9

Values represent percent of cells expressing both surface proteins. Data are presented as Mean ± SD.

### HIV‐1 infection is increased in stimulated cells obtained postexercise

**Figure 5:**
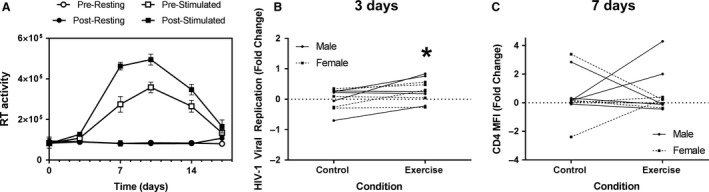
Susceptibility to HIV‐1 infection was increased in postexercise samples. (A) Example replication curve from one subject. Virus replication is presented as RT activity measured from cell culture supernatants (arbitrary units). (B) Plots show fold changes in HIV‐1 replication levels in the activated CD4^+^ T cells in each subject between control (0.02 ± 0.34) and exercise (0.29 ± 0.38) sessions at 3 days post infection. (C) The median fluorescence intensity (MFI) was determined for CD4 expression (Control: 0.46 ± 1.6, Exercise: 0.61 ± 1.5) following 3 days stimulation with anti‐CD3 and anti‐CD28. Data are presented as fold change from baseline and visualized as spaghetti plots with each line representing the change between paired samples; *n* = 10. *Indicates a statistically significant difference (*P* < 0.05) from the control session.

As an alternative means to measure the level of activation in CD4^+^ T cells, infection with the CXCR4‐tropic HIV‐1 NLX virus was chosen as a biological model system. HIV‐1 preferentially infects activated CD4^+^ cells due to the presence of increased metabolic activity. We hypothesized that increased activation in CD4^+^ cells harvested postexercise would lead to an increased susceptibility to HIV‐1 infection due to the increase in intracellular metabolism. To test this, we assayed virus replication in CD4^+^ cells isolated pre and post for both the exercise and control sessions for each participant. For each condition, we infected both unstimulated (resting) and cells pre‐activated with CD3/CD28/CD2 beads. The levels of virus replication were measured by quantification of RT activity in cell supernatants collected at various days post infection. Example data for one subject is shown in Figure 5A. Overall, infection of unstimulated, quiescent CD4^+^ T cells isolated from either the control or exercise session did not produce any significant amount of virus replication, nor were any differences between conditions observed. In activated CD4^+^ T cells, the RT activity peaked on average at 10 days post infection in cells from either the control or experiment sessions (Fig. 5A), then declined due to virus‐induced cell death. The average fold change in RT activity from pre‐to‐post exercise was calculated for each timepoint. At 3 days post infection, there was significantly greater levels of RT activity in the stimulated cells from the postexercise condition compared to resting controls (*P* = 0.03, Fig. 5B). Notably, there was no significant change in CD4 expression on cells isolated pre or post the control and exercise session at the time of viral infection (*P* = 0.84, Fig. 5C). The Bayesian threshold estimate indicates an 85.8% probability that the true parameter would be a greater than 10% increase in replication. Summary statistical data are shown in Table 1. The increased infection observed in cells obtained post exercise supports the hypothesis that there was increased metabolic activity in CD4^+^ T cells following acute bouts of resistance exercise.

### HIV‐1 latent infection in quiescent CD4^+^ T‐cells is not enhanced in cells isolated after an acute bout of resistance exercise

HIV‐1 preferentially infects activated CD4^+^ T cells, but it has been shown that HIV‐1 can infect quiescent cells partially activated in the G_1b_ phase of the cell cycle, or cells stimulated with various cytokines, including IL‐7 or IL‐13 (Unutmaz et al. 1999). These conditions are characterized by increased metabolic activity and levels of RNA expression equal to levels seen in the S phase of cell division (Korin and Zack 1998; Zack et al. 2013). Given that an acute bout of resistance exercise partially increased the activation state of CD4^+^ T cells in the absence of stimuli, we sought to investigate whether the cells isolated postexercise would support establishment of latent HIV‐1 infection. To assess this, the infected quiescent cells from both control and exercise sessions were reactivated at 14 dpi by treatment with CD3/CD28/CD2 activator beads and an additional RT sample collected 3 days post stimulation (17 dpi) to measure for latent virus infection. No significantly different levels of RT activity were observed between either the control or exercise session, suggesting that exercise does not stimulate CD4^+^ T cells sufficiently enough to support the establishment of HIV‐1 latency in CD4^+^ T cells (data not shown).

## Discussion

The present study examined the effect of resistance exercise on CD4^+^ T cell activation and utilized a viral model to indirectly assess intracellular metabolic activity via viral infection. The changes we observed in CD25 expression on non‐stimulated cells suggest that resistance exercise produces an effect on the activation state of CD4^+^ T cells in previously untrained individuals. Furthermore, the Bayesian analyses suggest a similar exercise‐induced increase in CD25 expression in cells responding to stimuli. Together, these findings indicate an increase in cell activation, which is also supported by the in vitro viral model showing a significant increase in viral infection in cells obtained postexercise.

Previous work from our laboratory using an acute bout of moderate intensity aerobic and resistance exercise demonstrated increased activation and proliferation of CD3^+^ cells following stimulation by either phytohemagglutinin (PHA) or co‐stimulation through CD28 (Siedlik et al. 2017). Field studies using elite athletes in competitive trials already demonstrate evidence of increased lymphocyte proliferation following bouts of aerobic exercise (Bassit et al. 2000; Tossige‐Gomes et al. 2014). To date, no studies have examined the effect of an acute bout of resistance training on T lymphocyte activation states and only a few have examined the effect of resistance training on lymphocyte proliferative capacity (Dohi et al. 2001; Koch et al. 2001; Potteiger et al. 2001). As an example, Potteiger et al. (2001) observed a statistically significant decrease in lymphocyte proliferation in untrained subjects following an acute bout of lower‐limb resistance training. Notably, however, this study along with the field studies mentioned above (Bassit et al. 2000; Tossige‐Gomes et al. 2014), quantified proliferation from mixed lymphocyte populations. Moreover, there was no assessment of either CD69 or CD25 expression which complicates any comparison with our results. Indeed, there is substantial ambiguity in the existing literature, which we propose is derived from variability in methods, including lymphocyte isolation protocols and the use of various mitogenic agents [Reviewed in (Siedlik et al. 2016; Campbell and Turner, 2018)]. In this project, we attempted to clarify some of the ambiguity of previous studies by focusing on a specific lymphocyte subset (CD4^+^ T cells) and utilizing a biological model of infection to assess exercise‐induced effects. The use of viral infection and replication as an outcome metric for immune function may seem counterintuitive, but reverse transcription and the process by which HIV integrates into the host genome is dependent upon the metabolic machinery of the cell, providing a unique physiological method to assess exercise‐induced alterations in CD4^+^ T cell metabolic state.

Cells isolated following exercise sessions supported enhanced replication of HIV‐1, but statistical significance was observed only at the second timepoint (3 dpi). This is likely because once infection is established, HIV‐1 has a logarithmic growth rate until the maximum threshold of virus replication is achieved (Ribeiro et al. 2010). In our experiments, the maximum threshold was typically achieved prior to the 10 dpi timepoint (overall mean = 9.575 ± 2.0 dpi). Given a constant MOI and no difference in cell surface expression of CD4 between the control and exercise conditions, the exponential growth rate of the viruses likely erased any differences in initial infection by 7 dpi. Nevertheless, the significant difference at 3 dpi demonstrated enhanced infection of the cells isolated postexercise. The significant increase in CD25 expression on nonstimulated cells obtained postexercise independently validated an increase in cellular activation. Combined, these data support the hypothesis that exercise induces an increase in the activation state of CD4^+^ T cells.

A primary limitation of this study, and of research in exercise immunology in general, centers on the external validity of the results themselves; namely, there is no direct translation from the in vitro assessments to an in vivo model. This is particularly relevant for the current project given the use of virus replication as an outcome measure. A previous study by Roubenoff et al. (1999) investigated alterations in viral RNA concentrations in plasma from HIV‐1 infected patients participating in a single bout of aerobic exercise. In their study, untrained, HIV‐1 positive individuals completed a 15 min, 60 cm vertical step test at a cadence of 1 step/sec. They found no significant increase in HIV RNA postexercise but speculated that the lack of change might have been due to the relatively high baseline viral loads in most of the subjects (Roubenoff et al. 1999). Similar to the replication threshold we observed at 10 dpi in vitro, the authors (Roubenoff et al. 1999) suspected there was a “ceiling effect,” or maximum threshold in replication, that limited exercise‐induced changes. Notably, patients with undetectable levels of HIV‐1 RNA at the start of the study showed increases in plasma HIV‐1 RNA levels postexercise. That result is consistent with the findings of the current study, despite the fact that we assayed virus replication in cells from healthy, uninfected individuals.

Compounding concerns related to in vitro versus in vivo models, the genetic diversity, environmental exposures, and health histories of individuals generate highly varied immune states (Tsang 2015). The innate variability in immune responses in human subjects tends to lead to less conclusive results compared to animal models, curbing enthusiasm for results obtained from human research (Davis 2008). The present study identified small, transient effects representative, in scale, of exercise‐induced changes in immune function. In an effort to embrace the observed variation (Gelman and Carlin 2017), the results of Bayesian analyses are provided to illustrate the probability of parameter values given the observed data. Bayesian methods provide a probability distribution for an estimate; meaning, we can assign a probability to our best estimate of the mean difference and all the possible values the parameter may take (Buchinsky and Chadha 2017). This Bayesian estimation provides more informative results about the magnitude of parameters and associated variance beyond that of traditional frequentist statistics (Kruschke 2013). Moreover, we believe that improved assessment and interpretation of variation in studies of human immune responses is needed as medicine shifts toward personalized, precision care (Tsang 2015; Collins and Varmus 2015).

Overall, these data support the hypothesis that an acute bout of resistance exercise in untrained individuals generates an increase in CD4^+^ T cell activation even in the absence of stimulation. We also demonstrated the use of an in vitro viral model to quantify a physiological effect, arguing for the use of other pathogens to investigate exercise‐induced changes in immune cell subsets. Infectious models, when combined with other stimulation assays, may help clarify the ambiguity in the current literature regarding the effect of exercise on immunity. It is our hope, that future studies using these designs will help advance translational research in exercise immunology and ultimately lead to better informed prescription of clinical exercise.

## Conflict of Interest

The authors report no conflict of interest.
